# Sensory profile of children with Central Auditory Processing Disorder (CAPD)

**DOI:** 10.1590/2317-1782/20212019282

**Published:** 2022-01-05

**Authors:** Flávia Regina Ribeiro Cavalcanti Buffone, Eliane Schochat

**Affiliations:** 1 Departamento de Terapia Ocupacional, Universidade Federal da Paraíba – UFPB - João Pessoa (PB), Brasil.; 2 Programa de Pós-graduação em Ciências da Reabilitação, Departamento de Fonoaudiologia, Fisioterapia e Terapia Ocupacional, Universidade de São Paulo – USP - São Paulo (SP), Brasil.

**Keywords:** Auditory Perception, Sensation, Child Development, Child, Neurodevelopmental Disorders

## Abstract

**Purpose:**

To analyze the sensory profile of children with auditory sensory processing disorder according to the Child Sensory Profile 2 and to verify potential associations between central auditory processing and sensory processing.

**Methods:**

Sixty children from two public schools in the city of João Pessoa, state of Paraíba, were evaluated. All children had their cognitive skills tested and their socioeconomic and demographic information collected. The children’s hearing, central auditory processing, and sensory processing were evaluated. SPSS Statistics version 25.0 was used for data analysis and the significant value adopted was 0.05. Descriptive analysis was performed using the central tendency method. The similarities among the test variables were measured by Student’s t-test and Mann-Whitney U test. The effect size (ES) between the groups was measured by Cohen’s d or Rosenthal’s r coefficient.

**Results:**

The average age of children with CAPD was 8.4 years, and their families had lower levels of income and education when compared to those without the disorder. Children with CAPD present more sensory differences than their peers with normative CAP. The Child Sensory Processing 2 results didn’t show any statistic associations with central auditory processing, and the effect size was of moderate magnitude for the visual system.

**Conclusion:**

Children with CAPD have more sensory differences than their peers according to the normative results of the Child Sensory Profile 2. An association between sensory and central auditory processing was not observed, except for the visual system.

## INTRODUCTION

Occupational therapist Anna Jean Ayres coined the term “sensory integration”, in the mid-fifties, after associating problems in sensory information processing in children regarded as clumsy with inadequate behavior or somewhat slower academic performance^(1)^. In 2007, in the context of contributing with more studies addressing this topic, Miller et al.^(2)^ suggested using the term “sensory processing” to rename the neurological functions of receiving, modulating, integrating, discriminating, and organizing sensory information received from the environment and the body itself regarding the different sensory systems. Interruption or inadequate functioning of any of these stages lead to sensory processing disorder (SPD), which can involve one or more sensory systems (touch, vestibular, proprioceptive, visual, auditory, gustatory, and olfactory), causing children to experience difficulties in performing their daily activities^(2,3)^.

Each individual responds to sensory information differently, and the presence of SPD is characterized by an imbalance between the neurobiological condition of processing and environmental sensory stimuli that interferes with the child’s occupational performance^(2-4)^. Studies on sensory processing and its functions aim to observe how an individual responds to the environmental demands to understand the associated implications for human behavior^(4)^.

Studies addressing sound issues have focused on characterizing sensory processing and on learning the influence of SPD on children’s functional performance. SPD has been found to influence sleep, motor coordination skills, behaviors of anxiety, food selectivity, and self-regulation, in addition to receptive and expressive language learning in autistic children, among others^(3-8)^. However, the actual influence of sensory processing on central auditory processing (CAP) remains unknown.

CAP is a function of the central auditory nervous system (CANS) that is responsible for sound perception and interpretation. Auditory processing encompasses the following set of skills: localization and lateralization of sound, auditory discrimination, auditory pattern recognition, temporal aspects of hearing, auditory performance, gradual decrease in auditory performance with competitive acoustic signals and with degraded acoustic signals^(9)^.

Thus, inadequate functioning of the CANS suggests the existence of a Central Auditory Processing Disorder (CAPD). CAPD is a deficit in the neural processing of the auditory stimulus whose symptoms include hearing difficulty in an acoustically unfavorable environment, which can be associated with other alterations or cause language and/or learning alterations, among other comorbidities^(10-12)^. Children with CAPD tend to experience academic difficulties and can be often characterized as distracted, forgetful, restless, talkative, and having difficulty with the concept of time^(10,11)^; therefore, they tend to ignore relevant auditory information. In a dynamic environment, the CANS functionality in these children is ineffective, and task performance oscillates^(9,10)^.

Despite the similarity in terms of the functional difficulties caused by both SPD and CAPD, little is known on the association between them. In a study conducted in 2011, Gavin et al.^(13)^ used electroencephalography to measure the evoked auditory potentials (N200 and P300) of 20 children with SPD and 71 with typical development aged between 5 and 10 years. The brain processing of auditory stimulus in the children with SPD proved significantly different from their counterparts. In addition, the children with SPD also presented lower amplitudes of P300, which generally occurs in children with developmental alterations.

Despite this information, little is known on the actual associations between SP and CAP. Aiming to clarify such relation, our goal is to analyze the sensory profile of children with CAPD and verify potential associations between CAP and SP. Our hypothesis is that children with CAPD develop more SPD than their counterparts with normal CAP.

## METHODS

This is an exploratory cross-sectional study that investigated the sensory profile of children with CAPD and the potential associations between AP and SP in a sample of 60 children aged between seven and 10 years and 11 months from two municipal public schools in João Pessoa, state of Paraíba. Our data are part of a more comprehensive project named “Central Auditory Processing, Sensory Processing, and Motor Coordination in Schoolchildren”, approved by the Research Ethics Committee of the University of São Paulo Medical School – protocol 1.856.907.

All participants were authorized by their parents or legal guardians by signing a Free and Informed Consent and formalized their acceptance by signing a Term of Consent.

To be included in the study, the children had to meet the following criteria:

- Study in the municipal school system, in one of the two public schools selected, and be regularly enrolled in classes from the 2^nd^ to the 5^th^ grade of elementary school;
- Be aged between seven and 10 years and 11 months at the time of the evaluation;
- Present a satisfactory cognitive level according to Raven's Colored Progressive Matrices (CPM) Test^(14)^.

The cases of children with genetic syndromes, congenital malformations, peripheral sensory deficiencies (visual and/or auditory), cognitive delay according to the CPM, intellectual disability, and neurological disorders, were excluded from this study.

All the selected children underwent the following assessments:

- Inspection of the external acoustic meatus for possible obstacles to the exam;
- Tonal audiometry aimed at selecting individuals with normal hearing acuity, i.e. hearing thresholds up to 20dB NA (ANSI 69) in the tonal audiometry and normal results considering the values established by Santos and Russo (1986) and Jerger (1970) in the vocal audiometry. Exam performed via airway at frequencies between 250 and 8000Hz;
- Immittance measures: consisting of (a) tympanometry and (b) acoustic reflex testing to assess middle ear function and the integrity of the stapedius muscle acoustic reflex. The inclusion criteria were: presence of type-A tympanometric curves and presence of ipsilateral acoustic reflexes for the frequencies of 500Hz, 1000Hz, and 2000Hz (Jerger,1970).

Only the children whose hearing tests were within the normal range and whose acoustic meatus had no alteration were subjected to the auditory processing assessments. All children underwent cognitive screening and a CAP assessment divided into two groups: 1) CAPD (children with alterations in at least two CAP tests) and 2) normal CAP (children without alterations or only one altered CAP test).

Two volunteer psychologists applied Raven's CPM^(14)^ in the cognitive screening of the eligible children, based on the Table XXV — CPM Standards for Public Schools. In turn, the percentile was classified according to the Table of Result Interpretation, varying from I to V, intellectually superior and intellectually deficient, respectively. Our study included the children classified between levels I and III (intellectually superior to intellectually average).

We obtained the data corresponding to the socioeconomic variables (age and gender of the children, age and educational level of parents or caregivers, and family income) by applying a form elaborated specifically for this research and filled out by the children’s parents or caregivers, which was used in the sample characterization.

The following devices were used during the hearing and CAP tests: acoustic booth, otoscope (Mark II 2.5V), immittance meter (Flute Plus – Inventis), digital audiometer (AVS 500 – Vibrasound), iPod Shuffle/mp3 (Apple) with the recorded AP test tracks, PAC equipment (PA400 – Acústica Orlandi), and headphones (TDH30). All equipment had been properly calibrated.

We applied the following behavioral tests in the AP assessment: 1) sound localization test in five directions^(15)^ to assess sound origin with normality reference of at least four hits; 2) Pediatric Speech Intelligibility (SPI)^(16)^ to assess background-figure of verbal sounds, using only the ipsilateral condition, signal/noise ratio of 0dB, -10dB, and -15dB – hits were scored when the ipsilateral competitive message was over 80%, 70%, and 60% of hits at the respective signal/noise ratios; 3) digit dichotic^(17)^ to verify binaural integration with normality criterion defined according to the child’s age: for those aged between 7 and 8 years, RE ≥ 85% of hits and LE ≥82% of hits, and for those aged between 9 and 10 years, RE ≥ 95% of hits and LE ≥95% of hits; 4) Random Gap Detection (RGDT)^(18)^ to assess temporal resolution, with the average of the four sound frequencies, ≤10ms, defined as the normality criterion for the children aged 7 or older.

All hearing and CAP assessments were performed by three speech therapists specialized in AP and the children who failed in at least two of the behavior tests were classified with altered CAP.

For the SP assessment, we used the Child Sensory Profile 2 (CSP2)^(2)^, which is a revised edition of the Child Sensory Profile aimed at verifying children’s sensory processing in everyday situations. The version used in this study was translated and adapted to the context of the Brazilian culture and is authorized by Pearson®; in addition, the main researcher was contacted.

The caregivers were asked to answer the questionnaire describing how each child responds to the different sensory stimuli throughout the day.

The behaviors are divided in sensory systems and sensory-based behaviors, and each item presents answers in a multiple-choice format composed of a Likert scale varying from zero to five: not applicable, almost never, occasionally, half of the time, frequently, and almost always, respectively^(2)^.

The responses are categorized in three major groups: quadrants, sensory section, and behavioral section.

Through the analysis of the quadrants, the CSP2 demonstrated how the children react to the different sensory stimuli in everyday life:

- Seeker (the child searches for sensory information in the environment);
- Avoider (the child avoids or cannot deal with sensory stimuli in the environment);
- Sensor (the child perceives more sensory information in everyday life);
- Bystander (the child does not perceive sensory stimuli in everyday life).

The sensory section in the questionnaire points to the sensory systems that potentially influence more the children’s routine and is grouped according to the types of sensory systems (auditory, visual, touch, movement, body position, and oral).

The behavioral section shows the influence of these different sensory stimuli’s response standards on the children’s behavior taking into consideration their behavioral, socioemotional, and attentional responses.

The raw score of the test is generated from the sum of the values in the items of each group listed above and transferred to a classification board of results that categorize the sensory processing of the groups into five different standards according to the normal distribution curve presented in the test: much less than others (-2 SD), less than others (-1 SD), just like the majority of others, more than others (+1 SD), and much more than others (+2 SD).

The CSP2 does not indicate whether the child has SPD, but it identifies children placed at the edges of the normality curve who may be facing difficulties in their occupational performance due to their sensory processing.

The sensory profile of children with CAPD was characterized according to the table of normative results, where those whose raw score dropped within the interval of the standard deviations (≤ -1 SD and ≥ +1 SD) in the normality curve were regarded as presenting a sensory difference.

Although the CSP2 is not a diagnostic test, it corresponds to a revised and updated version of the questionnaire that identify the children at the edge of the Gauss curve who are likely to be facing difficulties in everyday life associated with SPD.

The CSP2 was applied through an interview with the child’s caregiver conducted by two occupational therapists, properly trained research assistants. To indicate the answers, the caregiver was handed a visual scale with different color intensities corresponding to the Likert scale, which varied from “almost always” to “almost never.” After listening to each item, the caregiver pointed with their finger to the color representing the behavior closest to the child’s.

We inserted and tabulated the data on Excel for subsequent transfer to the SSPS Statistics software, version 25.0. The statistical significance value adopted was 5% (p ≤ 0.05).

We based the statistical analysis of the data on the sample of 60 individuals divided into two groups: the CAPD Group (n = 23) and the Normal CAP Group (n = 37). Sample characterization consisted of a descriptive analysis with central tendency measures.

We tested the normality assumption and applied Student’s t test (parametric) and Mann-Whitney’s U test (non-parametric) to compare the groups regarding the measures of central tendency and dispersion of the scores in the sensory processing tests. The effect size of the difference between the groups was measured by calculating the d (Cohen) or r (Rosenthal) coefficient.

## RESULTS

All children assessed were aged between 7 and 10 years and 11 months, while their parents or caregivers were aged 37.4 years old in average (SD = 12.5). Most of these families have an income per capita under R$ 364.40. Table 1 shows the sample characterization according to the CAP results. Among the 60 children in the sample, 23 (38.3%) were diagnosed with CAPD, whereas 37 (61.7%) belong to the normal CAP group. The children with CAPD were aged 8.4 months in average (SD = 0.9); the average age of the children with normal CAP increased to 9.3 years (SD = 1). Although the age difference between the groups does not have statistical relevance, we found that the older children tended to respond better to the CAP tests.

**Table 1:** Sample characterization for the 60 children studied

**Biological, socioeconomic, and population variables**	**CAPD**		**Normal CAP**		
	**Average**	**SD**	**Average**	**SD**	
Child’s age at assessment (years)	8.4	0.9	9.3	1	
	**N**	**%**	**N**	**%**	
**Gender**					
Male	10	43.5	18	48.6	
Female	13	56.5	19	51.4
**Per capita income (MW)**					
≤ ½	19	82.6	29	78.4	OR = 1.31
≥ ½	4	17.4	8	21.6	
**Caregiver’s educational level**					
≤ Elementary level	16	**69.6**	19	51.3	
High school or higher education	7	**30.4**	18	48.7	OR = 2.16

Caption: SD = Standard Deviation; OR = Odds Ratio; MW = Minimum Wage

Table 2 presents the measures of central tendency and dispersion of scores in the sensory processing tests according to the CAP results. We found no statistically significant difference between the groups. When considering the effect size (ES), the result of the visual processing (ES = 0.44) shows a difference of average magnitude between the normal CAP and CAPD groups for SP – visual system, the groups presented a slight difference in the remaining items in the CSP2.

**Table 2:** Descriptive values and comparative analysis between the groups for the scores in the sensory processing tests

	**Variable**	**CAP**	**n**	**Average**	**SD**	**Average**	**Min.**	**Max.**	**p**	**ES**
CHILDREN’S SENSORY PROFILE 2 (CSP2)	Seeker	Normal	37	48.78	19.22	49.00	5.00	94.00	0.79^a^	0.06^d^
		CAPD	23	50.04	15.81	50.00	22.00	84.00		
	Avoider	Normal	37	45.84	18.30	47.00	9.00	81.00	0.68^b^	0.05^r^
		CAPD	23	48.39	17.16	43.00	26.00	76.00		
	Sensor	Normal	37	43.05	14.54	43.00	17.00	83.00	0.52^a^	0.19^d^
		CAPD	23	45.78	18.27	51.00	18.00	76.00		
	Bystander	Normal	37	41.97	19.26	40.00	7.00	98.00	0.36^a^	0.25^d^
		CAPD	23	46.74	20.25	42.00	17.00	90.00		
	Auditory system	Normal	37	20.00	7.74	20.00	8.00	39.00	0.62^a^	0.13^d^
		CAPD	23	21.00	7.40	21.00	10.00	33.00		
	Visual system	Normal	37	14.86	5.77	15.00	6.00	29.00	0.13^a^	0.44^d^
		CAPD	23	17.39	7.02	18.00	4.00	30.00		
	Touch system	Normal	37	21.11	11.72	20.00	0.00	55.00	0.27^a^	0.30^d^
		CAPD	23	24.57	11.75	25.00	8.00	43.00		
	Movement	Normal	37	18.86	7.49	19.00	4.00	36.00	0.49^a^	0.20^d^
		CAPD	23	20.26	7.78	20.00	8.00	35.00		
	Body position	Normal	37	13.24	6.90	14.00	0.00	28.00	0.56^b^	0.07^r^
		CAPD	23	15.52	8.47	12.00	7.00	35.00		
	Oral sensory processing	Normal	37	23.24	9.96	22.00	0.00	45.00	0.65^a^	0.12^d^
		CAPD	23	24.43	10.26	26.00	7.00	42.00		
	Conduct	Normal	37	20.22	9.69	20.00	0.00	45.00	0.23^a^	0.30^d^
		CAPD	23	23.17	8.68	24.00	11.00	44.00		
	Socioemotional response	Normal	37	32.57	13.65	33.00	6.00	68.00	0.52^a^	0.17^d^
		CAPD	23	30.17	14.41	27.00	5.00	55.00		
	Attention	Normal	37	26.03	9.91	29.00	10.00	45.00	0.11^b^	0.20^r^
		CAPD	23	32.39	15.36	31.00	12.00	90.00		

Student’s t test for independent samples (^a^) and Mann-Whitney’s U Test (^b^)

Cohen’s effect size (d) and Rosenthal’s effect size (r)

Caption: SD = Standard Deviation; Min. = Minimum; Max. = Maximum; ES = Effect Size

**Figure 1:**
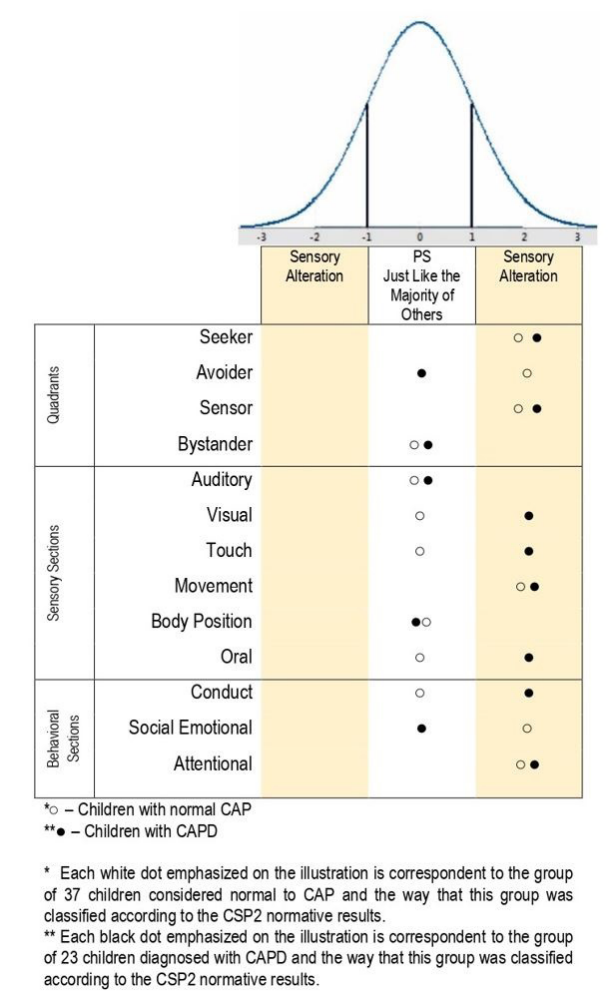
Characterization of the sensory profile of the groups of children with normal CAP and CAPD according to the CSP2 normative results

According to the normative result of the CSP2 (Figure 1), children with CAPD seem to have more sensory differences than those with normal CAP, as illustrated in Figure 1. It is worth highlighting that both the white and the black points in the image correspond to the group of 37 children with normal CAP and the group of 23 children with CAPD, respectively, i.e. each point corresponds to more than one child. In the group of children with CAPD, we observed sensory differences for 8 of the 13 results, as follows: in the quadrants, they appeared for the seeker and sensor; in the sensory section, they emerged in the visual, touch, movement, and oral systems; and in the behavioral section, they were found in the conduct and attention responses.

For the group of children with normal CAP, the sensory differences are present in 6 of the 13 results, as follows: in the quadrants, they appear for the seeker, avoider, and sensor; in the sensory section, for movement; and in the behavioral section, for the socioemotional and attention responses.

## DISCUSSION

According to the results presented in Table 1, the variable gender and the CAP results were not associated (OR = 0.81).

The average ages of the children with CAPD and normal CAP were similar to the findings of Vilela et al.^(19)^. Although the age difference between the groups was not statistically relevant, we found that older children tend to respond better to the CAP tests, as their CANS is more mature. Such finding is no different from the literature reports that demonstrate a positive influence of auditory neuromaturation on performance in PA behavioral tests^(20)^.

Barreira, Branco-Barreiro and Samelli^(21)^ found a different result with no age difference regarding the gap in the temporal resolution skill, which may have resulted from our different research choices. It is also possible that such AP skill matures at earlier ages.

All children in the sample are students from public schools. Most of them come from economically disadvantaged families, whose income per capita is below half of a minimum wage; therefore, both the studied groups – normal CAP and CAPD – belong to an economically vulnerable population. No statistical difference was observed between the income variable and the CAP results in our study; however, such condition may have influenced the increase in the CAPD index in the studied sample.

Souza and collaborators^(22)^ reported that the development of auditory skills is more damaged in children whose resources and stimuli are limited to the family environment. In general, these families lack interaction among its members, as many of them are too busy making the family’s living and thus have little time left to play or read with their children.

Even though all children in our sample come from public schools, the data collection procedure revealed that all parents from the families with better income (according to the answers to the socioeconomic and demographic questionnaire) are more participative and had better interaction with the evaluators, showing enhanced social and communication skills and more concern for the research as collaborators.

In general, these children whose caregivers have a low level of education normally belong to low-income populations. Neves and collaborators^(23)^ found that low-income families are constituted of parents with low level of education who, in turn, have children with growth problems and developmental cognitive and language levels below normal. Such information corroborated our result describing that the children whose caregivers have an education level equal or below elementary are more likely to develop CAPD (OR = 2.16). CAPD prevalence in this group reached the expressive value of approximately 70% (Table 1). The high CAPD prevalence in the studied population can be explained by the caregiver’s level of education. This result corroborated the findings of Souza and collaborators^(22)^, indicating that the children whose parents have better education present better results in cognitive and language development, since the children receive a higher quantity and quality of auditory and language stimuli, which allows for a more comprehensive auditory information experience. These parents tend to begin spontaneous communications with their children; in general, they talk more, read more, use plays and imaginary games, in addition to offering a more organized family environment that offers more resources.

As for the CSP2 results, the existence of an effect size of moderate magnitude between CAP and visual SP indicates an association between visual and auditory sensory information. Thus, we can infer that as the auditory information that reaches these children are not accurate, they make use of visual information as a complementary resource to perform their everyday life tasks, not only those related to school but all self-care and leisure activities. This can be related to the interconnection of sensory information through the neurological structures of the SNC and colliculus, a primarily visual structure, which contains a spatial auditory map with neurons from different regions that respond to a given auditory stimulus^(24)^.

Figure 1 shows the responses of the children with and without CAPD for the different items in the CSP2 according to the normative results of the test. In all of the eight results showing a sensory difference compared to the CSP2 normative population, the children with CAPD responded more to sensations.

The sensory differences in the visual, touch, movement, and sensory oral systems found in the children with CAPD may have derived from the multisensory integration that occurs in the CANS, i.e. upon failure, individuals automatically resort to the remaining sensory systems to compensate for the inefficiency of the auditory function^(24)^.

Such results confirm the hypothesis that children with CAPD present more sensory differences than their counterparts. A possible explanation is the manner in which the sensory information is integrated, since the sensory system works as a whole and failures in auditory processing interfere with how an individual uses, interprets, or reacts to the remaining environmental sensory stimuli. Lane and collaborators^(25)^ have recently published a study that confirms the theoretical background on sensory processing, pointing to the necessity of integrating information for an individual to perform daily tasks satisfactorily.

According to the CSP2, children’s behavior can be divided in quadrants, which correspond to a child’s behavior regarding the sensory information received. In this study, children with CAPD showed difference in two quadrants: seeker and sensor, which means that they search for and are sensitive to the visual, touch, movement, and oral stimuli more than most children.

Sensory sensitivity in children with CAPD is even higher than in those with normal CAP (Figure 1 and Table 1), which may be related to failures in their auditory perception that cause them to need greater attention to decode an auditory message successfully. Therefore, sensory information that should be irrelevant are more easily perceived by them, thus reflecting on their conduct and attention.

In this study, the CAP of children with CAPD also presented more sensory problems than normal children; however, the latter also presented sensory differences according to the CSP2 (Figure 1). These sensory differences can be associated with the sound behaviors observed while applying the research protocols and instruments in moments when the assessments needed to be interrupted for the child to deregulate their behavior and keep attentive to the task. Some of the children needed to stop, go to the bathroom, have a snack, or drink water.

Such behaviors can be associated with SPD. These children tend to have greater difficulty in performing everyday life tasks, especially school activities. According to Bar-Shalita, Vatine, and Parush^(26)^, problems in sensory modulation can interfere with performance, frequency, and pleasure involved in a given activity. Mimouni-Bloch^(27)^ also found problems in SP for the population of children with TDAH; however, the author did not establish any relation with CAPD.

In the children with normal CAP, 6 out of the 13 CSP2 results were altered (Figure 1), as follows: in the quadrants, for the items of seeker, sensor, and avoider; in the sensory section, only for movement; and the behaviors influenced by the sensory processing were socioemotional and attention.

We did not observe any differences between children with normal CAP and CAPD for the quadrants of seeker and sensor, movement and attention, which can be related to the similar environments occupied by these children. Most of the studied population live in risk areas of urban violence, being generally restricted to their household and school spaces. Since their houses are generally small and their schools have no parks, the need of movement that is peculiar to their age is often not satisfied and comes to be perceived as excessive.

Pedrosa, Caçola and Carvalhal^(28)^ conducted a study that demonstrated the influence of environmental factors on the sensory profile of 97 infants who attended daycare centers in Vila Real, Portugal. Although different from ours, the sample demonstrated the influence of the lack of quality stimuli in the environment on the infants’ sensory processing, as 11.3% and 22.7% showed at-risk or impaired sensory processing, respectively, according to the Test of Sensory Function in Infants. The study showed that the daycare center environment was associated with the babies’ sensory profiles; according to the authors, the space where the infants were assessed was negligent in terms of quantity and quality of toys allowing the infants to adequately develop their sensory-motor skills.

The scenario of the schools attended by the children in this study is no different from the daycare center in the aforementioned study. None of the environments have an outdoor area with toys to provide the children with a variety of games in a proper recreational space. Although the statistical association between sensory processing and the socioeconomic data in this study was not verified, the sensory differences found in the CSP2 of the children with normal CAP can be related to the lack of opportunity to experience proper environmental stimuli.

Román-Oyola and Reynolds^(29)^ found results similar to ours by analyzing the association between the socioeconomic conditions and the sensory profile of pre-school children from two educational institutions in Porto Rico. The sample studied consisted of 141 caregivers divided in two groups: 78 from an educational institution for low-income children supported by the Department of Health and Human Services of the United States, and 63 from private schools of a higher socioeconomic level. All of them answered a Short Sensory Profile and a questionnaire for demographic information. The results of the study indicated an association between sensitivity to movement and search for sensory information and the educational level of parents and family income. The scores of the children whose parents had better education were higher in these items than those of the children whose parents had a low educational level, i.e. they sought out movement stimuli more. The researchers also associated low socioeconomic levels with environmental conditions involving less resources and stimulation.

Despite such correlation, the population studied differs from the sample assessed in this study, especially regarding their sociocultural backgrounds. Thus, we suggest that further studies attempt to design the sensory profile of children whose families live in a socio-economically disadvantaged situation in Northeastern Brazil.

Another hypothesis to justify the search for movement in the group of children who did not present alterations is the physiological aspect at the child’s current developmental stage, as children at that age usually play and move around. Even though it is not necessarily a problem but often a pastime between activities, caregivers can be annoyed by such behavior, thus generating a false positive.

Bartie and collaborators^(30)^ analyzed the playing behavior of children from a low-income population in a community deep in South Africa. The children were monitored each afternoon for one hour after school for a week. The monitoring time was considered sufficient in a context of free and spontaneous play, even in the presence of the researcher. The children played games involving gross motricity activities, always in the company of a friend, relative, or adult, with a toy or another available object and used symbolically, among other activities, such as watching television. Even though the region where these children lived and played involved situations that offered risks to their safety, they were creative and took advantage of whatever resources were available in the environment. The researchers also observed that when switching from an activity to another, the children were always moving around, which corroborates the proposition that even in children with normal CAP, the behavior of seeking movement games can be considered normal for their age.

The sensory difference found in the sensitive and evasive quadrants for the children with normal CAP suggests that, as these children are not likely to present any alteration in oral and/or written language, they can inform their caregivers of any uncomfortable situations more easily. The same is not true for the children with CAPD, who often have difficulty in communicating a message clearly. This is only a hypothesis based on the results observed and reveals that much more is to be further explored in the scope of sensory processing.

## LIMITATIONS

Despite the relevance of our results, this study implies some important limitations:

- Due to its groundbreaking nature, it is composed of a convenience sample that is unrepresentative of the population;
- The education level and income of the studied sample may have influenced the results of the tests;
- The children’s age group varied from seven to 10 years and 11 months, which may have influenced the results of the CAP assessments.

## CONCLUSION

Children with CAPD have different SP characteristics than the CSP2 normative population. In the quadrants, differences appeared for the seeker and sensor, while in the sensory section, they presented in the visual, touch, movement, and oral systems. In the behavioral section, they are present in the conduct and attention response.

As for the association between CAP and SP, none of the CSP2 results indicated a statistical significance among the variables; however, th e effect size of the differences among them had a moderate magnitude for the visual system.

Even though it was not one of the goals of this study, the socioeconomic and demographic characteristics of the studied population seem to have influenced the CAP results, suggesting that such relation can be better explored in further studies.
