# Supplementary material for: Sensory profile of children with Central Auditory Processing Disorder (CAPD)
Source: Codas. 2022 Jan 5;34(1):e20190282. doi: 10.1590/2317-1782/20212019282 (PMC9886108; doi:10.1590/2317-1782/20212019282)
Supplement: Translation

# Translation: Perfil sensorial de crianças com Transtorno do Processamento Auditivo Central (TPAC)

**Authors:** Flávia Regina Ribeiro Cavalcanti Buffone, Eliane Schochat

**Affiliations:** 1 Departamento de Terapia Ocupacional, Universidade Federal da Paraíba – UFPB - João Pessoa (PB), Brasil.; 2 Programa de Pós-graduação em Ciências da Reabilitação, Departamento de Fonoaudiologia, Fisioterapia e Terapia Ocupacional, Universidade de São Paulo – USP - São Paulo (SP), Brasil.

**Keywords:** Percepção Auditiva, Sensação, Desenvolvimento Infantil, Criança, Transtornos do Neurodesenvolvimento

## Abstract

**Objetivo:**

Analisar o perfil sensorial de crianças com TPAC, de acordo com o Child Sensory Profile 2 e verificar possíveis associações entre o PAC e o PS.

**Método:**

Foram avaliadas 60 crianças em idade escolar de duas escolas públicas de João Pessoa, PB. Todas realizaram triagem cognitiva, tiveram suas informações socioeconômicas colhidas e foram submetidas às avaliações audiológica, do processamento auditivo central e do processamento sensorial. A análise dos dados foi feita com o SPSS Statistics, versão 25.0, e o valor de significância adotado foi de 0,05. Foi realizada a análise descritiva com medidas de tendência central. A associação entre as variáveis foi medida pelos testes t de Student e U de Mann-Whitney. O tamanho do efeito da diferença entre os grupos foi medido pelo coeficiente d de Cohen ou r de Rosenthal.

**Resultados:**

As crianças com TPAC tinham idade média de 8,4 anos, e suas famílias apresentaram renda e escolaridade inferior aquelas sem alteração do processamento auditivo. Foi observado que crianças com TPAC apresentam mais diferenças sensoriais que seus pares com PAC normal. Os resultados do Child Sensory Profile 2 não mostrou associação estatística com o processamento auditivo e a magnitude do tamanho do efeito entre as variáveis foi moderada para o sistema visual.

**Conclusão:**

Crianças com TPAC tem mais diferenças sensoriais que seus pares de acordo com o resultado normativo do Child Sensory Profile 2. Não foi observada associação entre o processamento sensorial e auditivo central, com exceção do sistema visual.

## INTRODUÇÃO

Integração sensorial foi o termo cunhado pela terapeuta ocupacional Anna Jean Ayres, em meados da década de 50, quando a então cientista associou problemas de processamento da informação sensorial em crianças consideradas desastradas, com comportamento inadequado ou mais lentas em relação ao aprendizado acadêmico^(1)^. Em 2007, Miller et al.^(2)^, levando em consideração o crescimento dos estudos em torno do tema, sugeriram a utilização do termo processamento sensorial, para nomear a função neurológica de receber, modular, integrar, discriminar e organizar as informações sensoriais recebidas pelo ambiente e pelo próprio corpo dos diferentes sistemas sensoriais. Quando alguma dessas etapas são interrompidas ou não funcionam adequadamente, o transtorno do processamento sensorial (TPS) acontece. Esse tipo de transtorno pode envolver um ou mais sistemas sensoriais (tátil, vestibular, proprioceptiva, visual, auditiva, gustativa e olfativa) e como consequência a criança pode ter dificuldades no desempenho de suas atividades cotidianas^(2,3)^.

A maneira como cada indivíduo responde as informações sensoriais é diferente e o TPS está presente quando há um desequilíbrio entre a condição neurobiológica do processamento e estímulos sensoriais no ambiente que interferem na performance ocupacional da criança^(2-4)^. Estudos sobre o processamento sensorial e suas funções procuraram observar como o indivíduo responde as demandas do ambiente, na tentativa de compreender suas implicações para o comportamento humano^(4)^.

Caracterizar o processamento sensorial e saber de que maneira o TPS repercute no desempenho funcional das crianças tem sido objeto de estudos de alguns pesquisadores. Já foi observado que o TPS influencia o sono, as habilidades de coordenação motora, os comportamentos de ansiedade, a seletividade alimentar, o desenvolvimento de comportamentos de autorregulação e de aprendizado de linguagem receptiva e expressiva em crianças autistas, entre outras^(3-8)^. Porém, é desconhecido até o momento de que maneira o processamento sensorial influencia o processamento auditivo central (PAC).

O PAC é uma função do sistema nervoso auditivo central (SNAC) responsável pela percepção e interpretação sonora. O processamento auditivo envolve uma série de habilidades que são: a localização e lateralização do som, a discriminação auditiva, o reconhecimento do padrão auditivo, os aspectos temporais da audição, a performance auditiva, a diminuição gradual da performance auditiva com sinais acústicos competitivos e com sinais acústicos degradados^(9)^.

Logo, quando o SNAC não funciona de maneira adequada, pode-se sugerir a existência de um transtorno do processamento auditivo central (TPAC). O TPAC é um déficit no processamento neural do estímulo auditivo e tem como um dos sintomas a dificuldade de ouvir em um ambiente acusticamente desfavorável, pode estar associado a outras alterações ou causar alterações de linguagem e/ou aprendizagem e outras comorbidades^(10-12)^. Cianças com TPAC tendem a ter dificuldades escolares e podem muitas vezes serem caracterizadas como distraídas, esquecidas, inquietas, falantes e apresentar dificuldade com o conceito de tempo^(10,11)^, por isso, elas tendem a ignorar informações auditivas relevantes. Num ambiente dinâmico, a funcionalidade do SNAC dessas crianças é ineficiente e há um desempenho variável da tarefa^(9,10)^.

Apesar das dificuldades funcionais ocasionadas pelo TPS e pelo TPAC serem semelhantes em alguns aspectos, pouco se sabe sobre a associação entre ambos. Em um estudo realizado em 2011, Gavin et al.^(13)^ utilizaram a eletroencefalografia para medir os potenciais evocados auditivos (N200 e P300) de 20 crianças com TPS e 71 com desenvolvimento normal com idades entre 5 e 10 anos. As crianças com TPS demonstraram um processamento cerebral do estímulo auditivo significativamente diferente dos seus pares. As crianças com TPS apresentaram menores amplitudes de P300, o que geralmente acontece com crianças com alterações do desenvolvimento.

Embora haja esse dado, pouco se sabe sobre as associações entre o PS e o PAC. Visando esclarecer essa relação, o objetivo desse artigo é analisar o perfil sensorial de crianças com TPAC e verificar as possíveis associações entre o PAC e o PS. A hipótese é que crianças com TPAC tem mais TPS que seus pares com o PAC normal.

## MÉTODO

Este é um estudo exploratório de corte transversal que investigou o perfil sensorial de crianças com TPAC e as possíveis associações entre o PA e o PS em uma amostra de 60 crianças entre sete e 10 anos e 11 meses de idade de duas escolas públicas municipais de João Pessoa, PB. Os dados aqui apresentados são parte de um projeto maior intitulado “Processamento Auditivo Central, Processamento Sensorial e Coordenação Motora de Crianças em Idade Escolar”, aprovado pelo comitê de ética em pesquisa da Faculdade de Medicina da Universidade de São Paulo sob o parecer 1.856.907.

Todos os participantes da pesquisa foram autorizados pelos seus pais ou responsáveis através da assinatura do Termo de Consentimento Livre e Esclarecido e formalizaram seu aceite através de sua assinatura no Termo de Assentimento.

Foram estabelecidos para o estudo os seguintes critérios de inclusão:

- Ser estudante da rede municipal de ensino de duas escolas públicas de ... e estar regularmente matriculado nas turmas do 2° ao 5° ano do ensino fundamental;

- Ter entre sete e 10 anos e 11 meses de idade no momento da avaliação;
- Apresentar nível cognitivo satisfatório de acordo com o Teste de Matrizes Coloridas de Raven (Coloured Progressive Matrices - CPM)^(14)^.

Crianças com síndromes genéticas, malformações congênitas, deficiências sensoriais periféricas (visual e/ou auditiva), com atraso cognitivo de acordo com o CPM, deficiência intelectual e distúrbios neurológicos foram excluídas da amostra.

Todas as crianças selecionadas para o estudo realizaram as seguintes avaliações:

- Inspeção do meato acústico externo para verificar possíveis impedimentos à realização do exame.
- Audiometria tonal: com o objetivo de selecionar os indivíduos com acuidade auditiva normal, ou seja, limiares de audibilidade até 20dB NA (ANSI 69) na audiometria tonal e resultados normais considerando os valores estabelecidos por Santos e Russo (1986) e Jerger (1970) na audiometria vocal. Foi realizada por via aérea, nas frequências de 250 a 8000Hz.
- Medidas de imitanciometria: compostas por (a) timpanometria e (b) pesquisa de reflexos acústicos para avaliar a função da orelha média e a integridade do reflexo acústico do músculo estapédio. Foi adotado como critério de inclusão a presença de curvas timpanométricas do tipo A e presença dos reflexos acústicos ipsilateral para as frequências de 500Hz, 1000Hz e 2000Hz (Jerger,1970).

Só realizaram as avaliações do processamento auditivo as crianças cujos testes audiológicos estavam dentro da normalidade e o meato acústico não apresentou qualquer alteração.

Todas as crianças foram submetidas a triagem cognitiva e a avaliação do PAC e dividida em dois grupos, 1) TPAC (aquelas que apresentaram alteração em pelo menos dois testes do PAC) e 2) PAC normal (crianças com nenhum ou apenas um teste do PAC alterado).

Para realizar a triagem cognitiva das crianças elegíveis para o estudo foi utilizado o CPM de Raven^(14)^ que foi aplicado por duas psicólogas voluntárias. Foi considerada como referência para o resultado a Tabela XXV — Normas do CPM para Escolas Públicas. O percentil, por sua vez, é classificado pela tabela de Interpretação dos Resultados, que varia de I a V, intelectualmente superior e intelectualmente deficiente, respectivamente. Foram incluídas nesse estudo as crianças classificadas entre os níveis I e III^-^ (intelectualmente superior a intelectualmente médio).

Os dados correspondentes às variaveis socioeconômicas (idade e sexo das crianças, idade e escolaridade dos responsáveis e renda familiar) foram obtidos através da aplicação de um formulário elaborado para pesquisa, respondido pelos pais ou responsáveis pela criança e utitlizados para a caracterização da amostra.

Para a realização dos testes audiológicos e de PAC foram utilizados os seguintes materias: a cabina acústica, o otoscópio (Mark II 2,5V), imitanciômetro (Flute Plus – Inventis), audiômetro digital (AVS 500- Vibrasom), iPod Shuffle/mp3 (Apple) com as faixas dos testes de PA gravadas, o equipamento do PAC (PA400 - Acústica Orlandi) e fones auriculares (TDH30). Todos os aparelhos estavam devidamente calibrados.

Os testes comportamentais utilizados para a avaliação do PA foram: 1) teste de localização sonora em cinco direções^(15)^, para avaliar a capacidade de origem do som, a referência de normalidade adotada foi de no mínimo quatro acertos; 2) *Pediatric Speech Inteligibility* (PSI)^(16)^, para avaliar a figura-fundo dos sons verbais, apenas a condição ipsilateral foi utilizada, a relação sinal/ruído foi de 0dB, -10dB e -15dB e foi considerado acerto quando a mensagem competitiva ipsilateral foi maior de 80%, 70% e 60% de acertos nas respectivas relações sinal/ruído; 3) dicótico de dígitos^(17)^, para verificar a integração binaural, o critério de normalidade utilizado estava de acordo com a idade das crianças do estudo, para crianças de 7 a 8 anos – OD ≥ 85% de acertos e OE ≥82% de acertos, para as idade de 9 a 10 anos - OD ≥ 95% de acertos e OE ≥95% de acertos; 4) *Random Gap Detection* (RGDT)^(18)^, para avaliar a resolução temporal, o critério de normalidade utilizado para crianças de sete anos ou mais para a média das quatro frequências sonoras foi de ≤10ms.

As avaliações audiológicas e de PAC foram realizadas por três fonoaudiólogas especialistas em PA e foram classificadas com PAC alterado as crianças que apresentaram falha em pelo menos dois dos testes de comportamento.

Para avaliar o PS foi utilizado o *Child Sensory Profile 2* (CSP2)^(2)^. O CSP2 é uma edição revisada do *Child Sensory Profile* e tem como objetivo verificar o processamento sensorial da criança nas situações do cotidiano. Nesse estudo foi utilizada sua versão traduzida e adaptada culturalmente para o Brasil, obtida a partir da autorização da Pearson® e contato direto com o pesquisador principal resposável pelo estudo.

Trata-se de um questionário aplicado aos responsáveis que registram a maneira como a criança responde aos diferentes estímulos sensoriais que ocorrem ao longo do dia.

Os comportamentos estão divididos por sistemas sensoriais e comportamentos de base sensorial e cada item apresenta uma possibilidade de resposta dentro de uma escala Likert que varia de zero a cinco, em que as opções são: não se aplica, quase nunca, ocasionalmente, metade das vezes, frequentemente e quase sempre, respectivamente^(2)^.

As respostas são categorizadas em três grandes grupos, quadrantes, seção sensorial e seção comportamental.

O CSP2 mostra através da análise da seção quadrantes, como as crianças reagem aos diferentes estímulos sensoriais existentes no cotidiano:

- buscador (quando a criança procura informações sensoriais no ambiente);
- evasivo (quando a criança evita ou não consegue lidar com os estímulos sensoriais do ambiente);
- sensível (quando a criança percebe mais as informações sensoriais do cotidiano);
- passivo (quando a criança não percebe os estímulos sensoriais existentes em sua rotina).

A seção sensorial do questionário aponta os possíveis sistemas sensoriais que exercem mais influência na rotina dessas crianças e está agrupada de acordo com os tipos de sistemas sensoriais existentes (auditivo, visual, tátil, de movimento, posição do corpo e oral).

Na seção comportamental pode-se verificar o quanto esses padrões de resposta diante dos diferentes estímulos sensoriais influenciam o comportamento da criança para as resposta de conduta, socioemocional e atencional.

O escore bruto do teste é feito a partir da somatória dos valores dos itens de cada grupo listado acima e transferido para o quadro de classificação dos resultados que categoriza o processamento sensorial dos grupos em cinco diferentes padrões de acordo com a curva de distribuição normal apresentada no teste: muito menos que os outros (-2 DP), menos que os outros (-1 DP), exatamente como os outros, mais que os outros (+1 DP) e muito mais que os outros (+2 DP).

O CSP2 não diz se a criança tem ou não um TPS, mas ele sugere que crianças nos extremos da curva de normalidade possam estar enfrentando dificuldades em seu desempenho ocupacional por consequência do seu processamento sensorial.

As crianças com TPAC tiveram seu perfil sensorial caracterizado de acordo com o quadro do resultado normativo do teste e aquelas cujo escore bruto caíram no intervalo dos desvios padrão (≤ -1 DP e ≥ +1 DP) da curva de normalidade foram categorizadas com diferença sensorial.

Apesar do CSP2 não ser um teste diagnóstico, ele é uma versão de questionário revisada e atual, capaz de sugerir que crianças que estão no extremo da curva de Gauss podem estar enfrentando dificuldades no cotidiano que estão associadas ao TPS.

O CSP2 foi aplicado por meio de entrevista ao acompanhante da criança, por duas terapeutas ocupacionais, auxiliares de pesquisa, devidamente treinadas. Para a marcação das resposta, foi entregue ao responsável, uma escala visual com diferentes intensidades de cores, para identificar a escala likert, que variou do quase sempre ao quase nunca. Assim que cada item era lido para o responsável, ele indicava com o dedo a cor que representava o comportamento mais aproximado da criança.

Os dados foram inseridos e tabulados no Excel e transportados para o software *SPSS Statistics*, versão 25.0. O valor de significância estatística adotado foi igual a 5% (p ≤ 0,05).

A análise estatística dos dados do estudo foi realizada usando como base a amostra de 60 indivíduos organizados em dois grupos: Grupo TPAC (n = 23) e Grupo PAC Normal (n = 37). Para caracterização da amostra foi realizada uma análise descritiva com medidas de tendência central.

O pressuposto de normalidade foi testado. Para comparar os grupos em relação às medidas de tendência central e de dispersão das pontuações nos testes de processamento sensorial foram utilizados os testes t de Student (paramétrico) e U de Mann-Whitney (não paramétrico). O tamanho do efeito da diferença entre os grupos foi medido por meio do cálculo dos coeficientes d (Cohen) ou r (Rosenthal).

## RESULTADOS

Todas as crianças avaliadas tinham idade entre 7 anos e 10 anos e 11 meses; seus responsáveis tinham uma média de 37,4 anos de idade(DP = 12,5). A maioria dessas famílias tem renda familiar per capita inferior a R$ 364,40. A Tabela 1 mostra a caracterização da amostra de acordo com o resultado do PAC. Das 60 crianças da amostra, 23 (38,3%) foram diagnosticadas com TPAC e 37 (61,7%) pertencem ao grupo com PAC normal. As crianças com TPAC tinham idade média de 8,4 meses (DP = 0,9); a média de idade das com PAC normal subiu para 9,3 anos (DP = 1). Embora a diferença de idade entre os grupos não tenha relevância estatística, observa-se que as crianças mais velhas tendem a responder melhor aos testes de PAC.

**Tabela 1:** Caracterização da amostra das 60 crianças estudadas

**Variáveis biológicas, socioeconômicas e demográficas**	**TPAC**		**PAC Normal**		
	**Média**	**DP**	**Média**	**DP**	
Idade da criança na avaliação (anos)	8,4	0,9	9,3	1	
	**N**	**%**	**N**	**%**	
**Sexo**					
Masculino	10	43,5	18	48,6	
Feminino	13	56,5	19	51,4
**Renda per capita (SM)**					
≤ ½	19	82,6	29	78,4	OR = 1,31
≥ ½	4	17,4	8	21,6	
**Escolaridade do responsável**					
≤ nível fundamental	16	**69,6**	19	51,3	
Nível médio e superior	7	**30,4**	18	48,7	OR = 2,16

Legenda: DP = Desvio padrão; OR = razão de chances; SM = Salário mínimo

A Tabela 2 apresenta as medidas de tendência central e de dispersão das pontuações nos testes de processamento sensorial de acordo com o resultado do PAC. Verifica-se que não houve diferença estatisticamente significante entre os grupos. Se levarmos em consideração o tamanho do efeito (TE), o resultado do processamento visual (TE = 0,44) mostra uma diferença de magnitude média entre os grupos PAC normal e TPAC para o PS –sistema visual, os demais itens do CSP2 tem uma diferença de magnitude fraca entre os grupos.

**Tabela 2:** Valores descritivos e análise comparativa dos grupos em relação às pontuações nos testes de processamento sensorial

	**Variável**	**PAC**	**n**	**Média**	**DP**	**Mediana**	**Mín.**	**Máx.**	**p**	**T.E.**
CHILD SENSORY PROFILE 2 (CSP2)	Buscador	Normal	37	48,78	19,22	49,00	5,00	94,00	0,79^a^	0,06^d^
		TPAC	23	50,04	15,81	50,00	22,00	84,00		
	Evasivo	Normal	37	45,84	18,30	47,00	9,00	81,00	0,68^b^	0,05^r^
		TPAC	23	48,39	17,16	43,00	26,00	76,00		
	Sensível	Normal	37	43,05	14,54	43,00	17,00	83,00	0,52^a^	0,19^d^
		TPAC	23	45,78	18,27	51,00	18,00	76,00		
	Passivo	Normal	37	41,97	19,26	40,00	7,00	98,00	0,36^a^	0,25^d^
		TPAC	23	46,74	20,25	42,00	17,00	90,00		
	Sistema auditivo	Normal	37	20,00	7,74	20,00	8,00	39,00	0,62^a^	0,13^d^
		TPAC	23	21,00	7,40	21,00	10,00	33,00		
	Sistema visual	Normal	37	14,86	5,77	15,00	6,00	29,00	0,13^a^	0,44^d^
		TPAC	23	17,39	7,02	18,00	4,00	30,00		
	Sistema tátil	Normal	37	21,11	11,72	20,00	0,00	55,00	0,27^a^	0,30^d^
		TPAC	23	24,57	11,75	25,00	8,00	43,00		
	Movimento	Normal	37	18,86	7,49	19,00	4,00	36,00	0,49^a^	0,20^d^
		TPAC	23	20,26	7,78	20,00	8,00	35,00		
	Posição do corpo	Normal	37	13,24	6,90	14,00	0,00	28,00	0,56^b^	0,07^r^
		TPAC	23	15,52	8,47	12,00	7,00	35,00		
	Processamento sensorial oral	Normal	37	23,24	9,96	22,00	0,00	45,00	0,65^a^	0,12^d^
		TPAC	23	24,43	10,26	26,00	7,00	42,00		
	Conduta	Normal	37	20,22	9,69	20,00	0,00	45,00	0,23^a^	0,30^d^
		TPAC	23	23,17	8,68	24,00	11,00	44,00		
	Resposta socioemocional	Normal	37	32,57	13,65	33,00	6,00	68,00	0,52^a^	0,17^d^
		TPAC	23	30,17	14,41	27,00	5,00	55,00		
	Atenção	Normal	37	26,03	9,91	29,00	10,00	45,00	0,11^b^	0,20^r^
		TPAC	23	32,39	15,36	31,00	12,00	90,00		

Teste t de Student para amostras independentes (^a^) e Teste U de Mann-Whitney (^b^)

Tamanho do efeito de Cohen (d) e Tamanho do efeito de Rosenthal (r)

Legenda: DP = Desvio padrão; Mín. = Mínimo; Máx. = Máximo; T.E. = Tamanho do efeito

De acordo com o resultado normativo do CSP2 (figura 1), crianças com TPAC parecem ter mais diferenças sensoriais que as que tem o PAC normal. A Figura 1 apresenta essas diferenças, cabe aqui ressaltar que os pontos brancos e pretos na figura correspondem ao grupo das 37 crianças com PAC normal e ao grupo das 23 crianças com TPAC respectivamente, ou seja, cada ponto corresponde a mais de uma criança. Entre o grupo de crianças com TPAC, foram observadas diferenças sensoriais para 8 dos 13 resultados. Nos quadrantes a diferença estava no buscador e sensível; na seção sensorial as diferenças foram para os sistemas visual, tátil, movimento e oral; e, na seção comportamental a conduta e atenção mostraram diferença.

**Figura 1:** Caracterização do perfil sensorial do grupo de crianças com PAC normal e TPAC de acordo com o resultado normativo do CSP2

Para o grupo de crianças com PAC normal, as diferenças sensoriais estão presentes em 6 dos 13 resultados: na seção dos quadrantes as diferenças apareceram para buscador, evasivo, sensível; na seção sensorial para movimento; e, na seção comportamental a resposta socioemocional e atencional.

## DISCUSSÃO

De acordo com o resultado apresentado na Tabela 1, não houve associação entre a variável gênero e o resultado do PAC (OR = 0,81).

As médias de idade das crianças com TPAC e PAC normal foram semelhantes as médias do estudo de Vilela et al.^(19)^. Embora as diferença de idade entre os grupos não tenha relevância estatística, observa-se que as crianças mais velhas tendem a responder melhor aos testes de PAC, uma vez que o SNAC está mais amadurecido. Essa informação não é diferente do encontrado na literatura que mostra a influência positiva da neuromaturação auditiva no desempenho dos testes comportamentais do PA^(20)^.

Barreira, Branco-Barreiro e Samelli^(21)^ encontraram um resultado diferente do apresentado e não observaram diferença de idade para detecção do *gap* na habilidade de resolução temporal. Isso pode ser resultado do teste de escolha ter sido diferente do utilizado na presente pesquisa, como também pela possibilidade dessa habilidade do PA amadurecer em idades mais precoces.

Todas as crianças da amostra são estudantes de escola pública. A maioria delas vive em famílias economicamente desfavorecidas, cuja renda *per capita* familiar foi inferior a meio salário mínimo, logo, ambos os grupos estudados, PAC normal e TPAC, pertencem a uma população economicamente vulnerável. Não foi observada diferença estatística entre a variável renda e o resultado do PAC neste estudo, porém essa pode ser uma condição que influenciou o aumento no índice de TPAC na amostra estudada.

Souza e colaboradores^(22)^ afirmam que crianças que têm recursos e estímulos limitados no ambiente familiar têm mais prejuízo no desenvolvimento de suas habilidades auditivas. São famílias que geralmente são carentes em relação a interação com seus membros, pois muitos deles estão tentando adquirir recursos para sobreviver, e, em sua maioria, têm pouca oportunidade de brincar ou ler para seus filhos.

Embora todas as crianças da presente pesquisa sejam de escolas públicas, uma observação realizada durante o procedimento de coleta de dados foi que os pais das famílias com melhor renda (de acordo com o respondido no questionário socioeconômico e demográfico) eram mais participativos e interagiam melhor com os avaliadores, mostrando melhores habilidades sociais e de comunicação, consequentemente, esses mesmos pais demonstraram mais preocupação em colaborar com o presente estudo.

Em geral, é na população de baixa renda que também estão as famílias com responsáveis de baixa escolaridade. Neves et al.^(23)^ verificaram que famílias de baixa renda e com pais de baixo nível de escolaridade têm crianças com problemas de crescimento e níveis de desenvolvimento cognitivo e de linguagem abaixo da normalidade. Essa informação corrobora com o resultado apresentado nesta pesquisa, em que crianças cujos responsáveis tem escolaridade igual ou menor que o ensino fundamental têm mais chance de ter o TPAC (OR = 2,16) prevalência do TPAC nesse grupo chegou a um valor expressivo de aproximadamente 70% (Tabela 1). A alta prevalência de TPAC na população estudada pode ser explicada pelo nível de escolaridade do responsável. Este resultado corrobora com o estudo de Souza et al.^(22)^, que diz que as crianças cujos pais têm mais escolaridade apresentaram melhores resultados de desenvolvimento cognitivo e de linguagem, pois a quantidade e a qualidade dos estímulos auditivos e de linguagem oferecidos às crianças são maiores, o que permite a ela uma maior experiência com a informação auditiva. Esses pais tendem a iniciar comunicações espontâneas com seus filhos; em geral, eles conversam mais, leem mais, utilizam brincadeiras e jogos imaginários, além de terem um ambiente familiar mais organizado e com mais recursos.

Em relação aos resultados do CSP2, a existência de um tamanho do efeito de magnitude moderada entre o PAC e PS visual mostra como as informações sensoriais visuais e auditivas estão associadas. Com isso, pode-se inferir que como a informação auditiva que chega até essas crianças não são precisas, elas utilizam a informação visual como recurso complementar para a realização de suas tarefas no cotidiano; compreende-se por tarefa não só as acadêmicas, mas as demais atividades de autocuidado e lazer. Isso pode acontecer porque as informações sensoriais se interconectam através das estruturas neurológicas do SNC e dos coliculos, estrutura primariamente visual, que contém um mapa espacial auditivo com seus neurônios de diferentes regiões que respondem ao estímulo auditivo^(24)^.

A Figura 1 mostra as respostas das crianças com e sem TPAC para os diferentes itens do CSP2, de acordo com o resultado normativo do teste. As crianças com TPAC tiveram oito resultados com diferença sensorial em relação a população normativa do CSP2 e em todos eles as crianças respondem mais as sensações.

As diferenças sensoriais dos sistemas visual, tátil, de movimento e sensorial oral encontradas nas crianças com TPAC podem ser consequência da integração multissensorial que acontece no SNAC, ou seja, quando essas falham, automaticamente os indivíduos recorrem aos demais sistemas sensoriais na tentativa de compensar a ineficiência da função auditiva^(24)^.

A partir desse resultado, pode-se confirmar a hipótese de que crianças com TPAC têm mais diferenças sensoriais que seus pares. Uma possibilidade é a forma como a integração das informações sensoriais ocorre, pois o sistema sensorial funciona como um todo, e falhas no processamento auditivo interferirão na maneira como o indivíduo usa, interpreta ou reage aos demais estímulos sensoriais do ambiente. Um artigo recente publicado por Lane et al.^(25)^ confirma o construto teórico do processamento sensorial em que afirma a necessidade da integração das informações para que um indivíduo tenha desempenho satisfatório na execução de suas tarefas cotidianas.

De acordo com o CSP2, o comportamento das crianças pode ser dividido em quadrantes, que é a maneira como a criança se comporta diante das informações sensoriais recebidas. No presente estudo, crianças com TPAC tiveram diferença em dois quadrantes: buscador e sensível. Isso quer dizer que essas crianças buscam e são sensíveis aos estímulos visuais, táteis, de movimento e orais mais que a maioria das crianças.

A sensibilidade sensorial nas crianças com TPAC é ainda maior que naquelas com PAC normal (Figura 1 e Tabela 1). Isso pode acontecer porque essas crianças têm falha na percepção auditiva e precisam estar mais alertas para tentar decodificar a mensagem auditiva. Com isso, informações sensoriais que deveriam ser irrelevantes são mais facilmente percebidas nessa população e isso tem resultado na conduta e atenção dessas crianças.

No presente estudo, as crianças com TPAC também apresentaram mais problemas sensoriais que as crianças normais para o PAC, mas essas últimas também tiveram diferenças sensoriais de acordo com o CSP2 (Figura 1). Essas diferenças sensoriais podem estar associadas a alguns comportamentos observados durante a aplicação dos protocolos e instrumentos de pesquisa, quando as avaliações precisavam ser interrompidas algumas vezes para que a criança autorregulasse o comportamento e conseguisse manter a atenção na tarefa a ser executada. Algumas delas precisavam parar, ir ao banheiro, fazer um lanche ou tomar um copo de água; outras, em número reduzido, precisaram ter a data da avaliação reagendada para concluir todos os procedimentos de pesquisa.

Esses comportamentos podem estar associados a um TPS. Essas crianças tendem a apresentar mais dificuldade em desempenhar suas tarefas de vida diária, em especial as escolares. Os problemas de modulação sensorial, segundo Bar-Shalita, Vatine e Parush^(26)^, podem interferir no desempenho, na frequência e na sensação de prazer de uma atividade realizada. Problemas no PS também foram encontrados por Mimouni-Bloch^(27)^ na população de crianças com TDAH, porém nenhuma relação foi feita com o TPAC.

Nas crianças com PAC normal, 6 dos 13 resultados do CSP2 estavam alterados (Figura 1). Na seção dos quadrantes, os itens buscador, sensível e evasivo; na seção sensorial, o movimento foi o único com diferença; os comportamentos influenciados pelo processamento sensorial foram o socioemocional e o de atenção.

Não foi observada diferença em crianças com PAC normal e TPAC para o quadrante de buscador e sensível, movimento e atenção. Isso pode ser explicado pela semelhança existente no ambiente que essas crianças estão inseridas. A maioria da população estudada reside em área de risco para violência urbana. São crianças que estão geralmente restritas ao espaço de casa e da escola, e como suas casas geralmente são pequenas e as escolas não possuem parque, a necessidade de brincadeiras de movimento características dessa idade muitas vezes não encontra espaço e é percebida como excessiva.

Um estudo realizado por Pedrosa, Caçola e Carvalhal^(28)^ verificou como os fatores ambientais podem influenciar o perfil sensorial de 97 lactentes frequentadores de creches em Vila Real, Portugal. Apesar da amostra ser diferente da apresentada no presente estudo, eles verificaram o quanto a escassez de estímulos de qualidade no ambiente pode interferir no processamento sensorial dos lactentes e viram que 11,3% e 22,7% tiveram processamento sensorial de risco e deficiente, respectivamente, de acordo com o *Test of Sensory Function in Infants.* O ambiente da creche esteve associado ao perfil sensorial do bebê, e o espaço onde crianças foram avaliadas é, segundo os autores, negligenciado em relação à quantidade e à qualidade de brinquedos que permitem aos lactentes um desenvolvimento sensório-motor adequado.

Se levarmos em consideração as escolas que as crianças deste estudo estão inseridas, elas não diferem da situação encontrada na creche do estudo supracitado. Ambos os ambientes não oferecem área externa com brinquedos que permitam às crianças, durante o recreio, realizar uma variedade de brincadeiras em espaço apropriado. Embora a associação estatística entre o processamento sensorial e os dados socioeconômicos desse estudo não tenha sido verificada, as diferenças sensoriais encontradas no CSP2 das crianças com PAC normal podem estar associadas à falta de oportunidade de estímulos ambientais adequados.

Resultado semelhante ao presente estudo foi encontrado por Román-Oyola e Reynolds^(29)^, que verificaram a associação entre as condições socioeconômicas e o perfil sensorial de crianças do pré-escolar de duas instituições de ensino de Porto Rico. A amostra estudada foi composta por 141 responsáveis pelas crianças, que estavam divididas em dois grupos: 78 de um centro de estudo para crianças de baixa renda, que recebe apoio do Departamento de Saúde e Serviços Humanos dos Estados Unidos, e 63 responsáveis por crianças de escolas privadas de nível socioeconômico mais alto. Todos responderam o *Short Sensory Profile* e um questionário com informações demográficas. O resultado do estudo mostrou associação entre a sensibilidade ao movimento e a busca sensorial com o nível de educação dos pais e a renda familiar. As crianças cujos pais tinham mais escolaridade pontuaram mais nesses itens que as de pais com baixa escolaridade, ou seja, buscaram mais estímulos de movimento. Os pesquisadores também associaram o baixo nível socioeconômico a condições de ambiente com menos recursos e estimulação.

Apesar de realizar essa correlação, a população estudada difere da avaliada no presente estudo, principalmente em relação aos aspectos socioculturais. Logo, sugere-se que novos estudos sejam feitos procurando traçar o perfil sensorial de crianças cujas famílias vivem em situação socioeconômica desfavorecida no nordeste do Brasil.

Outra hipótese para a busca por movimento no grupo de crianças sem alteração pode ser o aspecto fisiológico no momento atual do desenvolvimento da criança, pois crianças dessa faixa etária brincam e se movimentam. Isso não necessariamente é um problema, muitas vezes é um passatempo entre uma atividade e outra, mas aos olhos dos responsáveis esse comportamento pode incomodar, resultando em um falso positivo.

O comportamento do brincar de crianças numa população de baixa renda de uma comunidade no interior da África do Sul foi avaliado por Bartie e colaboradores^(30)^. Nesse estudo, as crianças foram acompanhadas por uma semana, diariamente, à tarde, durante uma hora após as aulas. O tempo de observação das crianças foi considerado suficiente quando o brincar livre e espontâneo aconteceu, mesmo na presença do pesquisador. As brincadeiras realizadas pelas crianças envolveram atividades de motricidade grossa, sempre na companhia de um amigo, membro da família ou adulto, com algum brinquedo ou objeto disponível utilizado de maneira simbólica, e outras atividades, como assistir televisão. Embora a região que as crianças residiam e brincavam tivesse questões que colocavam em risco sua segurança, elas eram criativas e utilizavam como recurso o que havia disponível no ambiente. Os pesquisadores também observaram que quando essas crianças estavam transitando entre uma atividade e outra elas permaneciam em constante movimento. Esse resultado corrobora com a proposição feita, de que mesmo nas crianças com PAC normal a procura por brincadeiras de movimento pode ser um comportamento considerado normal para a idade.

A diferença sensorial encontrada nos quadrantes sensível e evasivo das crianças com PAC normal sugere que, como provavelmente essas crianças não têm alteração da linguagem oral e/ou escrita, elas podem ter mais facilidade para reportar aos seus responsáveis a situação de desconforto. Isso não acontece com as crianças com TPAC, que muitas vezes têm dificuldade de transmitir a mensagem de forma clara. Essa é apenas uma hipótese com base no resultado encontrado, e mostra que muito ainda precisa ser explorado em relação ao estudo do processamento sensorial.

## LIMITAÇÕES

Apesar da relevância dos resultados encontrados nesse estudo, ele apresenta limitações importantes:

- por ser um estudo pioneiro, ele foi realizado com uma amostra de conveniência e pouco representativa da população;
- o nível de escolaridade e renda da amostra estudada pode ter influenciado no resultado dos testes;
- a faixa etária das crianças variou dos sete aos 10 anos e 11 meses e essa diferença de idade pode ter influenciado nos resultados das avaliações do PAC.

## CONCLUSÃO

A característica do PS de crianças com TPAC é diferente quando comparada a população normativa do CSP2. Para os quadrantes a diferença apareceu em buscador e sensível; na seção sensorial essa diferença apareceu para os sistemas visual, tátil, movimento e oral; e, na seção comportamental a conduta e resposta atencional mostrou diferença.

Em relação a associação entre o PAC e PS, nenhum dos resultados do CSP2 mostrou significância estatística entre as variáveis, porém o tamanho do efeito da diferença entre elas foi de magnitude moderada para o sistema visual.

Apesar de não ser um objetivo do estudo, as características socioeconômicas e demográficas da população estudada pareceram ter influência nos resultados do PAC, o que sugere que tal relação possa ser melhor explorada em estudos futuros.
